# Neuropathologic Correlates of Hippocampal Atrophy in the Elderly: A Clinical, Pathologic, Postmortem MRI Study

**DOI:** 10.1371/journal.pone.0026286

**Published:** 2011-10-17

**Authors:** Robert J. Dawe, David A. Bennett, Julie A. Schneider, Konstantinos Arfanakis

**Affiliations:** 1 Department of Biomedical Engineering, Illinois Institute of Technology, Chicago, Illinois, United States of America; 2 Rush Alzheimer's Disease Center, Rush University Medical Center, Chicago, Illinois, United States of America; National Institutes of Health, United States of America

## Abstract

The volume of the hippocampus measured with structural magnetic resonance imaging (MRI) is increasingly used as a biomarker for Alzheimer's disease (AD). However, the neuropathologic basis of structural MRI changes in the hippocampus in the elderly has not been directly assessed. Postmortem MRI of the aging human brain, combined with histopathology, could be an important tool to address this issue. Therefore, this study combined postmortem MRI and histopathology in 100 elderly subjects from the Rush Memory and Aging Project and the Religious Orders Study. First, to validate the information contained in postmortem MRI data, we tested the hypothesis that postmortem hippocampal volume is smaller in subjects with clinically diagnosed Alzheimer's disease compared to subjects with mild or no cognitive impairment, as observed in antemortem imaging studies. Subsequently, the relations of postmortem hippocampal volume to AD pathology, Lewy bodies, amyloid angiopathy, gross infarcts, microscopic infarcts, and hippocampal sclerosis were examined. It was demonstrated that hippocampal volume was smaller in persons with a clinical diagnosis of AD compared to those with no cognitive impairment (*P* = 2.6×10^−7^) or mild cognitive impairment (*P* = 9.6×10^−7^). Additionally, hippocampal volume was related to multiple cognitive abilities assessed proximate to death, with its strongest association with episodic memory. Among all pathologies investigated, the most significant factors related to lower hippocampal volume were shown to be AD pathology (*P* = 0.0018) and hippocampal sclerosis (*P* = 4.2×10^−7^). Shape analysis allowed for visualization of the hippocampal regions most associated with volume loss for each of these two pathologies. Overall, this investigation confirmed the relation of hippocampal volume measured postmortem to clinical diagnosis of AD and measures of cognition, and concluded that both AD pathology and hippocampal sclerosis affect hippocampal volume in old age, though the impacts of each pathology on the shape of the hippocampus may differ.

## Introduction

Postmortem MRI of the human brain followed by histology can be used to better understand the neurobiologic basis of in vivo imaging findings in Alzheimer's disease (AD) [1], [2] and other conditions [3], [4]. Combining antemortem MRI data with histopathologic information requires that the time between imaging and death is minimized to eliminate changes that may occur in the brain in the time between the two events [2]. By employing postmortem MRI, brain changes between imaging and histology are minimized. Thus, the combination of postmortem MRI and histology may be a cost effective experimental approach for investigating the neuropathologic basis of imaging findings in the elderly. Further enhancing the utility of postmortem MRI in research on aging is the fact that living elderly patients are often uncooperative during scans, increasing the chance for bulk head motion and image artifacts.

The medial temporal lobe (MTL) has been the focus of much research due to its important role in the development of dementias, including AD. Several studies have used MRI to measure the volume of MTL structures, especially the hippocampus, in vivo [2], [5]–[12]. These measurements have been compared across groups of subjects with a clinical diagnosis of possible or probable AD, mild cognitive impairment (MCI), and no cognitive impairment (NCI). Typically, lower hippocampal volumes were shown to be associated with possible or probable AD and MCI. Fewer studies have examined the MTL using postmortem MRI [13]–[17]. A study at 7.0-T found a significant reduction in the cross-sectional area of the hippocampus in 13 cases of histopathologically confirmed AD versus controls [15]. A pair of studies found associations of postmortem hippocampal volume measurements with AD neuropathology [14] and also with antemortem measures of delayed recall performance [17]. Finally, a study that employed a rating scale of medial temporal lobe atrophy found a significant link between atrophy, AD, and other types of neuropathology [16]. However, none of the aforementioned postmortem studies investigated in detail the potential effects of several different neuropathologies on the quantitative volume or shape of the hippocampus using a large number of MRI and histopathology datasets, limiting their ability to attribute hippocampal volume loss to AD and not any other comorbid pathology.

AD is not the only type of neuropathology that has been linked to reduction of hippocampal volume. Hippocampal sclerosis (HS) is characterized by severe neuronal loss with gliosis in the cornu ammonis 1 (CA1) subregion of the hippocampus [18], [19]. Interestingly, a recent report alludes to the possibility that the effect of HS on hippocampal volume may not be noticeable in subjects who also have a high amount of AD pathology [2]. Therefore, current diagnostic methods may not be able to distinguish AD from HS in elderly subjects, either on the basis of hippocampal volume or the patient's neuropsychological profile (both conditions involve similar types of dementia) [20], [21].

The purpose of this study was two-fold. The first goal was to verify that postmortem MRI volumetry of the human hippocampus captures useful information related to the subject's antemortem clinical diagnosis and cognition, and, therefore, may be a valuable tool for research on aging, despite the changes that occur in brain tissue as a result of decomposition, extraction from the cranium, and fixation. Therefore, we tested the hypothesis that the volume of the hippocampus, assessed with postmortem MR volumetry, is lower for clinically diagnosed AD subjects compared to MCI and NCI subjects, and lower for MCI subjects compared to NCI subjects, as previously demonstrated by in vivo studies [22], [23]. We also tested the hypothesis that hippocampal volume measured postmortem is positively related to performance in each of five different cognitive domains (episodic memory, semantic memory, working memory, perceptual speed, and visuospatial ability). From in vivo studies, we anticipated a positive relationship between episodic memory and postmortem hippocampal volume [11], [17], [24], which would further corroborate the important role of postmortem MRI measurements of hippocampal volume. Following the initial verification segment of this study, the second goal was to use postmortem MRI and histopathology to investigate the relation of hippocampal volume to neuropathologies frequently occurring in the elderly: AD pathology, Lewy bodies, amyloid angiopathy, gross infarcts, microscopic infarcts, and HS pathology. We expected postmortem hippocampal volume to be most affected by AD and HS, with a smaller effect related to amyloid angiopathy [2]. Shape analysis based on spherical harmonics was also employed to visualize potential differences in the shape and size of the hippocampus in the presence of different pathologies. The unique combination of postmortem MRI volumetry and histology employed in this study allowed us to investigate correlates of hippocampal structural changes based on reliable measures of different types of neuropathology that are common in old age.

## Methods

### Ethics Statement

Cerebral hemispheres were obtained from deceased elderly subjects who were participants in either of two longitudinal clinical-pathologic studies of aging: the Rush University Memory and Aging Project (MAP) and the Religious Orders Study (ROS) [25]. All participants provided written informed consent and signed an anatomical gift act. The study was approved by the Institutional Review Board of Rush University Medical Center. All clinical investigation has been conducted according to the principles expressed in the Declaration of Helsinki.

### Subjects

During 18 months of investigation, 100 individuals from the MAP and ROS projects who died with a final diagnosis of AD (n = 44), MCI (n = 24), or NCI (n = 32) proximate to death were included in this study. The clinical classification was rendered by an expert neurologist and was based on the review of all years of clinical data, including annual neuropsychologic testing that was reviewed by a board-certified clinical neuropsychologist, and antemortem clinical evaluations that included a medical history and neurological examination from all years prior to death (without access to neuropathologic or postmortem neuroimaging data). The clinical diagnosis of AD was based on the criteria of the joint working group of the National Institute of Neurological and Communicative Disorders and Stroke and the Alzheimer's Disease and Related Disorders Association (NINCDS/ADRDA) as previously described [26]. The clinical diagnosis of MCI referred to persons who had cognitive impairment but did not meet clinical criteria for dementia [27], [28]. Since clinical diagnoses of MCI and AD for both the ROS and MAP cohorts were made according to identical criteria by the same group of investigators, data was merged for all analyses [29].

### Assessment of Cognitive Performance

The two longitudinal studies (MAP and ROS) have 19 cognitive tests in common. From those, the Mini-Mental State Examination (MMSE) is used for descriptive purposes, and one other test, the complex ideas auditory comprehension subtest from the Boston Diagnostic Aphasia Exam, is only used in diagnostic classification. The remaining 17 tests assess a broad range of cognitive abilities. The scores from individual tests are converted to z-scores based on the mean and standard deviation at baseline, and are combined to obtain measures of five cognitive domains. Briefly, episodic memory is evaluated with seven tests including immediate and delayed recall of story A from Logical Memory and of the East Boston Story, and Word List Memory, Recall, and Recognition from the Consortium to Establish a Registry for AD (CERAD). Semantic memory is assessed with three tests including a 15-item version of the Boston Naming Test, Verbal Fluency, and a 10-item reading test. Working memory is also assessed with three tests, including Digit Span Forward and Backward and Digit Ordering. There are two tests of perceptual speed, including Symbol Digit Modalities Test, and Number Comparison. Finally, there are two tests of visuospatial ability, including a 15-item version of Judgment of Line Orientation and a 9-item version of Standard Progressive Matrices. Also, a global cognitive score is formed as a composite of all 17 test scores. Details of the cognitive tests and the use of combined measures from both cohorts have been previously described [25], [30], [31].

### Brain Hemisphere Preparation

After a subject's death, an autopsy technician removed the brain and dura from the calvarium by severing the cranial nerves and the spinal cord at the level of the foramen magnum. Immediately following removal of the intact brain, the cerebrum was separated from the cerebellum and brainstem by cutting through the cerebral peduncles rostrally to the mammillary bodies. The cerebrum was then divided into left and right hemispheres by bisecting the corpus callosum. The hemisphere with visible pathology was selected for imaging. If both hemispheres had similar amounts of visible pathology or no visible pathology, the selection was arbitrary. The chosen hemisphere was immersed in phosphate-buffered 4% formaldehyde solution (prepared from paraformaldehyde) and refrigerated at 4°C in a sealed plastic container within 30 minutes after removal from the skull. While in storage, the 4% formaldehyde solution was changed weekly.

### Image Acquisition

Scans took place, on average, after 62 days of fixation (range 22–200). All scans were performed using a 3.0-T GE MRI scanner (General Electric, Waukesha, WI), with the hemisphere immersed in room temperature formaldehyde solution in a clear, sealed acrylic container. Since high spatial resolution MRI scans were performed in this study, appropriate measures were taken to reduce the transmission of vibrations from the MRI scanner to the brain hemispheres. Specifically, the hemispheres were gently anchored in place using netting, and the acrylic container did not make direct contact with the vibrating scanner due to the use of a specially designed bridge apparatus [32]. A 2D fast spin-echo sequence with two echo times was used to acquire proton density weighted (PD-weighted) and *T_2_*-weighted images, in sagittal slices through the hemispheres, using the following parameters: TR = 3.6 s, TE_1_ = 13.0 ms, TE_2_ = 52.0 ms, echo-train length = 6, FOV = 16 × 16 cm, slice thickness = 1.5 mm, acquisition matrix = 256×256 zero-padded to 512×512, acquired 6 times and averaged (NEX = 6). The total scan time was 31 minutes.

### Image Analysis

**Figure 1:**
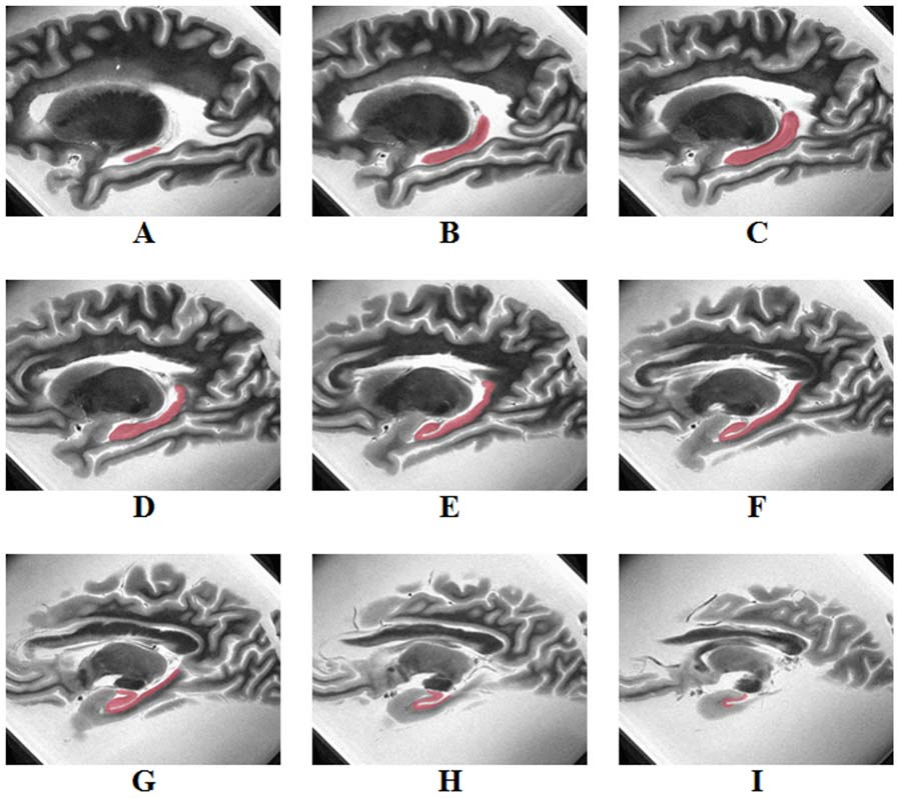
A typical three-dimensional region of interest created by manually outlining the hippocampal formation in consecutive T_2_-weighted sagittal images of a human cerebral hemisphere. Image A corresponds to the most lateral slice and image I to the most medial slice. The hemisphere remained immersed in formaldehyde solution during the MRI scan. In-plane resolution was 0.3125×0.3125 mm^2^ after zero-padding of the k-space acquisition matrix.

A single operator, blinded to all cognitive data and diagnostic classifications, outlined the hippocampal formation in every *T_2_*-weighted sagittal slice for each hemisphere, to generate a 3D region of interest (ROI), using landmarks that have been described previously [33]. The sagittal plane was used as the primary ROI selection view because of its superior in-plane resolution (0.625×0.625 mm^2^ originally and 0.3125×0.3125 mm^2^ after zero-filling in k-space), and the coronal and axial views were referenced as necessary to confirm landmark position. The intraobserver reliability of this ROI selection method was assessed using the union overlap metric (intersection of two ROIs divided by their union) in a subsample of 30 hippocampi which were outlined twice by the same observer. The hippocampal ROI included all subdivisions of the cornu ammonis (CA), the dentate gyrus, and the subiculum (Fig. 1). The volume of each ROI was calculated by multiplying the number of voxels within the ROI by a volume of 0.146 mm^3^ per voxel. The volume of the entire hemisphere was calculated using a similar procedure, but with automatic rather than manual segmentation methods.

### Neuropathological Diagnosis

Following imaging, the cerebral hemisphere was placed in a Plexiglas jig and cut coronally into 1-cm thick slabs. Slabs underwent complete macroscopic evaluation and dissection of diagnostic blocks, including midfrontal, middle temporal, inferior parietal cortex, entorhinal cortex, hippocampus, anterior basal ganglia, anterior thalamus, and midbrain, including substantia nigra. These regions were embedded in paraffin, cut into sections, and mounted on glass slides. Brain autopsy procedures have been described previously [34], [35].

Neuropathologic diagnoses were made by a board-certified neuropathologist blinded to age and all clinical data (JAS). Bielschowsky silver stain was used to visualize neuritic plaques and neurofibrillary tangles in the frontal, temporal, parietal, entorhinal cortex, and the hippocampus. A neuropathologic diagnosis of low, intermediate, or high likelihood AD was made based on the Braak score for neurofibrillary pathology and the CERAD estimate of neuritic plaques, as recommended by the NIA-Reagan criteria [36]. Details of the pathologic diagnoses of AD have been described previously [34], [35]. In this study, the NIA-Reagan score was transformed into a dichotomous variable by forming one group from the no AD and low likelihood subjects (non-AD) and another group from the intermediate and high likelihood subjects (AD). Lewy bodies were identified with antibodies to alpha-synuclein [34] and were classified as nigral, limbic, or neocortical based on recommended guidelines [37]. Cerebral amyloid angiopathy (CAA pathology) was assessed on 20 micron paraffin-embedded sections from five brain regions: four neocortical regions comprising the midfrontal (Brodmann area, BA, 46/9), inferior temporal (BA20), angular gyrus (BA39), and calcarine cortices (BA17), and one medial temporal region, the hippocampus (BA24). Sections were stained with antibodies to amyloid ß protein (Clone 6F/3D, M 0872, DAKO, 1∶100; or 10D5, courtesy of Elan pharmaceuticals) using a standard protocol [38], [39]. We used a semiquantitative grading system from 0 (none) to 5 (most severe) for each region and computed the average for the overall brain. For analyses, scores of less than 0.5 were classified as minimal or no CAA, scores between 0.5 and 2.5 were classified as mild to moderate CAA, and scores above 2.5 were classified as moderately severe to very severe CAA [39]. The brain and slabs were examined for cerebral infarcts (infarcts visualized by the naked eye) as previously described [37]. For analyses, we classified cases according to the number of gross chronic (old) infarcts: none, only one, or more than one. Microscopic infarcts (defined as infarcts seen microscopically but not macroscopically) were identified on 6-micron hematoxylin and eosin-stained routine sections [40]. Cases were classified according to the number of chronic microscopic infarcts: none, only one, or more than one. HS was assessed on 6 micron sections of hippocampus and was defined as substantial neuronal loss and gliosis including the CA1 sector.

### Statistical Analysis

Analysis of covariance (ANCOVA) was used to test for differences in hippocampal volume (dependent variable) across the clinically diagnosed AD, MCI, and NCI groups. The following covariates were also examined: total hemisphere volume, age, sex, education, hemisphere side (left or right hemisphere), specimen source (MAP or ROS), the duration between death and immersion in formaldehyde (postmortem interval to fixation, PMI_f_), the duration between death and MRI (postmortem interval to imaging, PMI_i_), and lag between last cognitive exam and death. The covariates that were not significant were removed one by one such that all of the parameters remaining in the final ANCOVA model were significant. We used *P*<0.05 as the criterion for statistical significance in these and all other statistical tests throughout the study.

To evaluate the relation of postmortem hippocampal volume to antemortem cognition, we used multiple regression analysis with volume as the dependent variable and cognitive score as the independent variable. Separate regressions were first carried out for the global cognitive score and each of the five individual domain scores (episodic, semantic, and working memory, perceptual speed, and visuospatial ability), while controlling for the previously mentioned covariates. Subsequently, all five domain scores were entered in a combined regression model to determine which were the most significant predictors of hippocampal volume. In the regression models, non-significant terms were eliminated one by one until all of the remaining model coefficients were significant.

ANCOVA was then used to examine the relations of AD and other common types of pathology to hippocampal volume. Each type of pathology was investigated individually at first, while controlling for the previously mentioned covariates. Subsequently, all types of pathology were entered in a combined model in order to determine which were the most significant predictors of hippocampal volume. In these ANCOVA models, non-significant factors were eliminated from the model one by one until all of the remaining factors were significant.

The Spherical Harmonic-Point Distribution Model (SPHARM-PDM) Toolbox [41] was employed for analysis of the hippocampal shape associated with each pathology that was found to be significantly related to hippocampal volume. A three-dimensional mesh (a networked collection of points) was first produced to represent the surface of each hippocampal ROI. All left hippocampi were mirrored to appear as right. A spherical parameterization was then computed for each mesh. Group differences in the distances between corresponding points on the surface meshes were detected using the Hotelling T^2^ metric [42]. In controlling for multiple comparisons, a false discovery rate of 5% was accepted.

## Results

### MAP and ROS subjects and hemispheres

Demographic information, clinical diagnoses, and other characteristics for the 65 MAP subjects and 35 ROS subjects are shown in Table 1. The average age at death for all 100 subjects was 87.8±6.0 years (mean ± s.d.). MAP subjects were older than their ROS counterparts at the time of death (3.9% older, *P* = 0.0065) and had fewer years of education (18% fewer, *P* = 2.4×10^−5^), but no other statistically significant differences between the groups' demographic characteristics, including incidence of clinically diagnosed MCI and AD, or neuropathologically diagnosed AD, were observed. The time between a subject's last cognitive examination and death averaged 249±142 days (mean ± s.d.) for the 100 hemispheres in this study, and was not statistically different between the MAP and ROS hemispheres. The PMI_f_ averaged 7.2±4.5 hours (mean ± s.d.) for the 100 hemispheres in this study and was not statistically different between the MAP and ROS hemispheres. The PMI_i_ averaged 62±36 days, and was significantly shorter for MAP hemispheres compared to ROS hemispheres (22% shorter, *P* = 0.032, Table 1). This difference was due to delays in transportation of some ROS hemispheres from satellite autopsy locations to the central imaging site.

**Table 1:** Selected demographic, clinical, and neuropathologic characteristics of subjects from the Memory and Aging Project (MAP) and the Religious Orders Study (ROS) included in this work.

Characteristics	MAP	ROS	All
N	65	35	100
Age at death, y (SD)	a89.0 (5.7)	85.6 (5.9)	87.8 (6.0)
Male, n (%)	24 (37%)	12 (34%)	36 (36%)
Education, y (SD)	b15.0 (3.2)	18.3 (4.1)	16.2 (3.9)
Height, in. (SD)	65.0 (3.8)	64.4 (4.8)	64.8 (4.2)
Left hemisphere, n (%)	35 (54%)	17 (49%)	52 (52%)
Mini-mental state examination score proximate to death (SD)	20.6 (9.1)	20.7 (10.2)	20.6 (9.4)
Global cognition z-score (SD)	−0.99 (1.21)	−0.89 (1.31)	−0.95 (1.24)
Hippocampal volume, cm^3^ (SD)	2.67 (0.59)	2.64 (0.63)	2.66 (0.60)
Postmortem interval to fixation, h (SD)	6.8 (4.2)	8.1 (5.0)	7.2 (4.5)
Postmortem interval to imaging, days (SD)	c56 (29)	72 (44)	62 (36)
Days between last cognitive exam and death (SD)	252 (140)	243 (148)	249 (142)
Clinical diagnosis, n (%)	-	-	-
–No cognitive impairment	22 (34%)	10 (29%)	32 (32%)
–Mild cognitive impairment	14 (21%)	10 (29%)	24 (24%)
–Alzheimer's disease	29 (45%)	15 (43%)	44 (44%)
Neuropathological diagnosis of Alzheimer's disease, n (%)	-	-	-
–No	33 (51%)	18 (51%)	51 (51%)
–Yes	32 (49%)	17 (49%)	49 (49%)

a Significantly different from ROS (*P* = 0.0065).

b Significantly different from ROS (*P* = 2.4×10^−5^).

c Significantly different from ROS (*P* = 0.032).

### NCI, MCI, and AD subjects and hemispheres

Characteristics of NCI, MCI, and AD subjects are shown in Table 2. Among the three groups, there were no significant differences in age, sex, education, hemisphere side, PMI_f_, PMI_i_, or the time between the last cognitive exam and death, according to simple ANOVA with Tukey's honestly significant difference post hoc test accounting for multiple comparisons. The AD group was characterized by significantly lower MMSE scores and cognitive domain z-scores than the MCI and NCI groups (*P*<10^−8^ for all). In addition, the MMSE and cognitive domain z-scores were all lower for the MCI group compared to the NCI group, but this difference only reached the level of statistical significance for the perceptual speed domain (*P* = 0.040). Total hemisphere volume was significantly smaller in the MCI group compared to the NCI group (8% smaller, *P* = 0.012). The hemisphere volume was also smaller in the AD group compared to NCI (7% smaller, *P* = 0.015).

**Table 2:** Selected characteristics of groups of subjects with no cognitive impairment (NCI) and clinical diagnoses of mild cognitive impairment (MCI) and Alzheimer's disease (AD).

Characteristics	NCI	MCI	AD
N	32	24	44
Age at death, y (SD)	86.4 (6.1)	87.8 (7.3)	88.8 (4.9)
Male, n (%)	11 (34%)	6 (25%)	19 (43%)
Education, y (SD)	15.5 (3.7)	16.0 (4.5)	16.8 (3.6)
Height, in. (SD)	65.2 (3.8)	63.7 (5.0)	65.1 (3.9)
Left hemisphere, n (%)	17 (53%)	12 (50%)	23 (52%)
Days between last cognitive exam and death (SD)	263 (146)	270 (152)	227 (133)
MMSE proximate to death (SD)	28.0 (1.7)	25.5 (4.0)	d12.6 (8.7)
Global cognition (SD)	0.08 (0.48)	−0.37 (0.59)	d−2.03 (1.01)
Episodic memory (SD)	0.25 (0.45)	−0.23 (0.88)	d−2.08 (1.07)
Semantic memory (SD)	0.05 (0.58)	−0.22 (0.73)	d−2.04 (1.65)
Working memory (SD)	0.07 (0.74)	−0.37 (0.68)	d−1.57 (1.12)
Perceptual speed (SD)	−0.28 (0.84)	e−0.91 (0.87)	d−2.21 (1.03)
Visuospatial ability (SD)	−0.04 (0.48)	−0.37 (0.59)	d−2.02 (1.01)
Hippocampal volume, cm^3^ (SD)	3.01 (0.45)	2.85 (0.50)	f2.30 (0.55)
Total hemisphere volume, cm^3^ (SD)	g428.5 (45.4)	392.9 (44.5)	398.9 (44.4)
Postmortem interval to fixation, h (SD)	7.4 (4.8)	8.4 (5.1)	6.5 (3.9)
Postmortem interval to imaging, days (SD)	64 (40)	58 (34)	62 (34)

d Significantly different from NCI and from MCI (*P*<10^−8^ for both).

e Significantly different from NCI (*P* = 0.040).

f Significantly different from NCI (*P* = 2.6×10^−7^) and from MCI (*P* = 9.6×10^−7^).

g Significantly different from MCI (*P* = 0.012) and from AD (*P* = 0.015).

### Relation of hippocampal volume measured postmortem to clinical diagnosis

**Figure 2:**
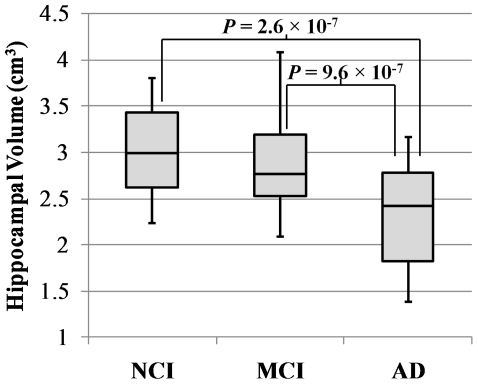
Box plot of hippocampal volumes for clinically diagnosed Alzheimer's disease, mild cognitive impairment, and no cognitive impairment subjects. The horizontal line through each box indicates the median. The ends of each box indicate the 25^th^ and 75^th^ percentile locations, and the lines indicate the range of the data. Hippocampal volume was significantly smaller in Alzheimer's disease than in cases of mild cognitive impairment or no cognitive impairment.

The intraobserver reliability of the ROI selection technique was high, as evidenced by the 88% union overlap that was calculated using a subsample of 30 subjects. By ANCOVA, postmortem hippocampal volume was significantly lower in the group of clinically diagnosed AD subjects compared to the MCI (21% lower, *P* = 9.6×10^−7^) and NCI (21% lower, *P* = 2.6×10^−7^) groups (Fig. 2). There was no significant difference in hippocampal volume between the MCI and NCI groups (*P* = 0.96). Similar findings resulted from ANCOVA that did not control for hemisphere volume. Similar findings also resulted from ANCOVA in which subject height, which others have used as a surrogate for total intracranial volume [24], replaced the hemisphere volume covariate (total intracranial volumes were not available since the brain hemispheres were not imaged in situ).

### Relation of hippocampal volume measured postmortem to cognition

**Figure 3:**
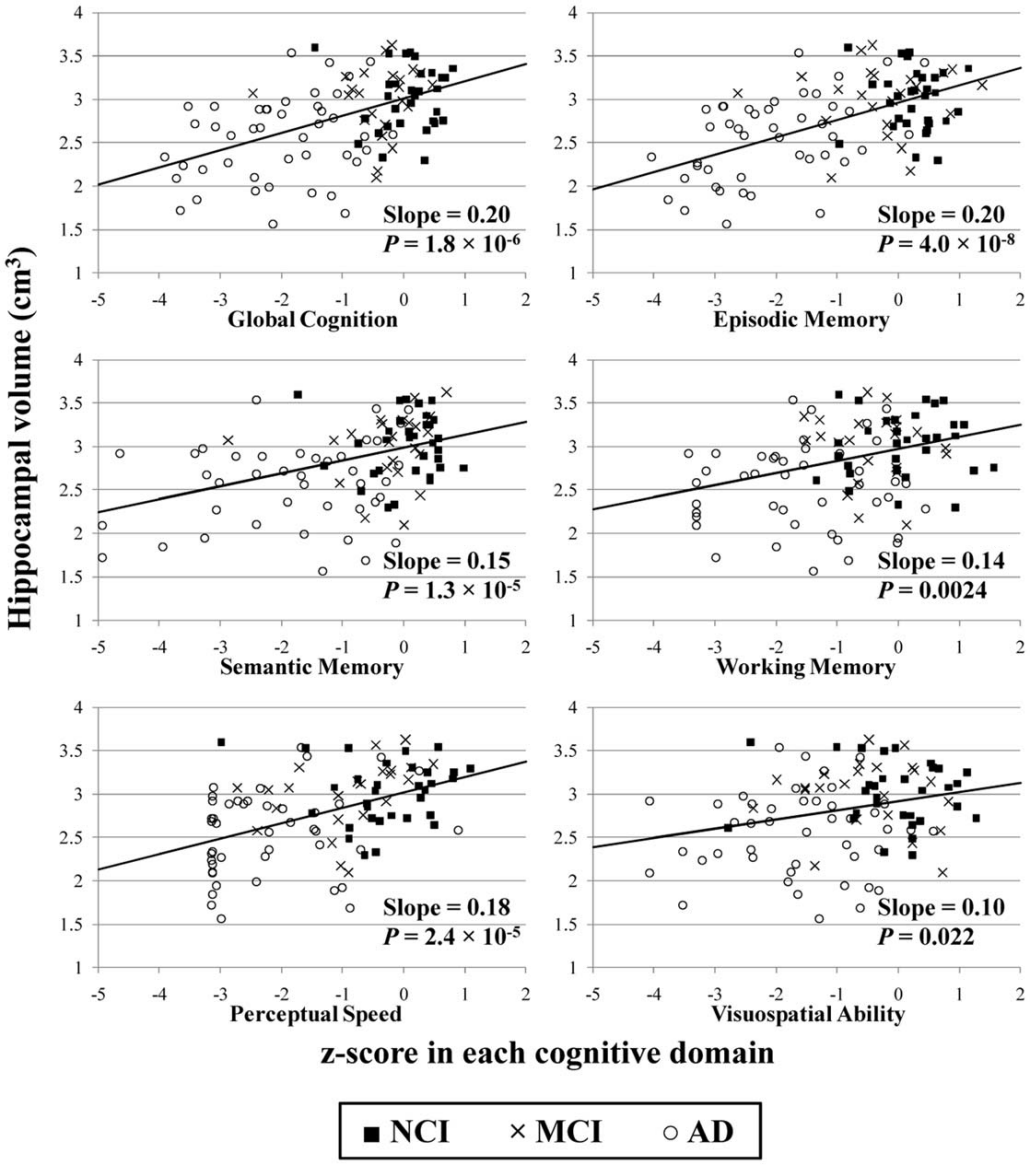
Plots of the hippocampal volume as a function of the z-score for global cognition, episodic memory, semantic memory, working memory, perceptual speed, and visuospatial ability. Clinical diagnosis for each subject is indicated by marker type.

The relation of hippocampal volume measured postmortem to level of cognition was investigated next. Linear regressions revealed a statistically significant positive relationship between hippocampal volume and global cognition (Table 3, Fig. 3). Separate linear regressions also revealed significant positive relationships between hippocampal volume and each of the five individual cognitive domains: episodic memory, semantic memory, working memory, perceptual speed, and visuospatial ability (Table 3, Fig. 3). When all five individual cognitive scores were entered in a single combined regression, only episodic memory retained a statistically significant relation to hippocampal volume. After removal of those terms that were not significant, this combined model became equivalent to the individual regression of hippocampal volume against episodic memory (Table 3). Results were similar when hemisphere volume was not included in the regressions and also when subject height replaced hemisphere volume in the regressions.

**Table 3:** Coefficient values for linear regression models of postmortem hippocampal volume as a function of global cognition and other covariates, and the corresponding P-values.

Factor	Regression Coefficient	P-value
Global cognition	**0.20**	1.8×10^−6^
Episodic memory	**0.20**	4.0×10^−8^
Semantic memory	**0.15**	1.3×10^−5^
Working memory	**0.14**	0.0024
Perceptual speed	**0.18**	2.4×10^−5^
Visuospatial ability	**0.10**	0.022

Regression coefficients significantly different from zero in boldface.

All models adjusted for total hemisphere volume, age, sex, education, hemisphere side, specimen source, the postmortem interval to fixation, the postmortem interval to imaging, and time between last cognitive exam and death.

### Relation of hippocampal volume measured postmortem to neuropathology

We next examined the relation of hippocampal volume to AD and other neuropathologies that are frequently found in the aging human brain. When an individual ANCOVA was executed for each type of pathology, HS showed the strongest relation with hippocampal volume, both in terms of magnitude (−0.80 cm^3^) and its statistical significance (*P* = 1.2×10^−7^) (Table 4). AD pathology also had a large and highly significant relation with hippocampal volume (−0.38 cm^3^, *P* = 4.1×10^−4^). The relation of moderately severe to very severe amyloid angiopathy was also significant (−0.41 cm^3^, *P* = 0.0020). There was a marginally significant relation of hippocampal volume with both nigral (−0.52 cm^3^, *P* = 0.059) and limbic (−0.38 cm^3^, *P* = 0.051) Lewy bodies.

**Table 4:** Effects of different types of neuropathology on hippocampal volume, assessed individually using analysis of covariance, and the corresponding P-values.

Type of Pathology	Number of Subjects	Association with Hippocampal Volume (cm^3^)	P-value
Alzheimer's (NIA criteria)	49	**−0.38**	4.1×10^−4^
Lewy bodies	-	**-**	-
–Nigral	4	−0.52	0.059
–Limbic	9	−0.38	0.051
–Neocortical	8	−0.29	0.15
Amyloid angiopathy	-	-	-
–Mild to moderate	36	**−**0.01	0.94
–Moderately severe to very severe	35	**−0.41**	0.0020
Gross infarcts	-	-	-
–Only one	16	0.11	0.46
–More than one	11	−0.16	0.35
Microscopic infarcts	-	-	-
–Only one	24	−0.17	0.19
–More than one	4	−0.43	0.13
Hippocampal sclerosis	13	**−0.80**	1.2×10^−7^

Effects significantly different from zero in boldface.

All models adjusted for total hemisphere volume, age, sex, education, hemisphere side, specimen source, postmortem interval to fixation, and postmortem interval to imaging.

All types of neuropathology measured in this study were then entered in a combined ANCOVA model. After the least significant factors were eliminated from the model one by one, AD pathology (−0.29 cm^3^, *P* = 0.0018) and HS (−0.73 cm^3^, *P* = 4.2×10^−7^) were the only pathologies that retained statistically significant relations to hippocampal volume (Table 5). Dichotomizing the Lewy body, gross infarct, and microscopic infarcts factors (pathology present or not present, regardless of quantity) also did not substantially alter the results, nor were these factors found to be significant. Removing hemisphere volume from the analysis did not substantially alter the results, nor did replacing hemisphere volume with subject height.

**Table 5:** Effect of AD pathology and hippocampal sclerosis on hippocampal volume, assessed simultaneously using analysis of covariance, and the corresponding P-values.

Type of Pathology	Number of Subjects	Effect on Hippocampal Volume (cm^3^)	P-value
Alzheimer's (NIA criteria)	49	**−0.29**	0.0018
Hippocampal sclerosis	13	**−0.73**	4.2×10^−7^

Only statistically significant neuropathologies were included in the final combined analysis of covariance model.

Model adjusted for total hemisphere volume, age, sex, education, hemisphere side, specimen source, postmortem interval to fixation, and postmortem interval to imaging.

The strong associations between hippocampal volume, AD, and HS pathology were investigated further. First, an interaction factor between AD and HS was added to the ANCOVA model. The addition of this term did not substantially alter the results, nor was the interaction factor found to be significant in the ANCOVA model. Next, subjects were compared by groups that had been histopathologically diagnosed as having neither AD nor HS, AD but not HS, HS but not AD, or both AD and HS (Table 6). AD subjects (without HS) were shown by ANOVA (no correction for hemisphere volume or any other parameters) to have hippocampal volumes that were approximately 0.35 cm^3^ smaller than the non-AD, non-HS subjects (*P* = 0.0068). AD subjects with concomitant HS had hippocampal volumes that were 0.74 cm^3^ smaller than the AD-only subjects (*P* = 5.8×10^−4^) and 1.09 cm^3^ smaller than the non-AD, non-HS subjects (*P* = 1.5×10^−7^). Similarly, HS-only subjects had hippocampal volumes that were 0.72 cm^3^ smaller than the AD-only subjects (*P* = 0.034) and 1.07 cm^3^ smaller than the non-AD, non-HS subjects (*P* = 4.1×10^−4^).

**Table 6:** Selected demographic, clinical, and neuropathologic characteristics of subjects with no histopathologically diagnosed Alzheimer's disease (AD) or hippocampal sclerosis (HS), with AD only, with HS only, and with both AD and HS.

Characteristics	Non-AD, Non-HS	AD	HS	AD+HS
N	47	40	4	9
Age at death, y (SD)	86.8 (7.0)	88.7 (4.9)	87.7 (7.8)	88.8 (3.3)
Male, n (%)	14 (30%)	18 (45%)	1 (25%)	3 (33%)
Education, y (SD)	15.3 (3.7)	16.8 (4.1)	16.3 (2.9)	18.0 (3.2)
Height, in. (SD)	64.0 (4.3)	65.6 (3.9)	63.3 (4.3)	65.8 (4.0)
Left hemisphere, n (%)	25 (53%)	20 (50%)	3 (75%)	4 (44%)
Mini-mental state examination score (SD)	24.8 (6.0)	h18.4 (10.3)	h12.9 (13.3)	h12.6 (9.1)
Global cognition (SD)	−0.39 (0.88)	h−1.21 (1.24)	−1.69 (1.53)	h ^,^ i−2.40 (1.19)
Episodic memory (SD)	−0.18 (0.98)	−1.27 (1.35)	−1.51 (1.51)	−2.61 (1.02)
Semantic memory (SD)	−0.30 (0.98)	−1.17 (1.45)	−1.77 (2.15)	−2.83 (2.25)
Working memory (SD)	−0.41 (0.97)	−0.89 (1.20)	−1.55 (1.43)	−1.63 (1.35)
Perceptual speed (SD)	−0.82 (1.14)	−1.43 (1.23)	−2.16 (1.16)	−2.61 (0.86)
Visuospatial ability (SD)	−0.59 (1.09)	−0.89 (1.21)	−1.95 (1.86)	−1.46 (1.33)
Total hemisphere volume, cm^3^ (SD)	409.8 (44.3)	410.8 (47.6)	368.3 (13.8)	391.8 (59.9)
Hippocampal volume, cm^3^ (SD)	2.94 (0.51)	h2.59 (0.48)	h ^,^ i1.87 (0.40)	h ^,^ i1.85 (0.47)
Postmortem interval to fixation, h (SD)	7.7 (5.1)	6.7 (4.1)	6.2 (2.9)	7.9 (3.5)
Postmortem interval to imaging, days (SD)	68 (42)	54 (29)	45 (22)	71 (22)
Days between cognitive exam and death (SD)	253 (144)	243 (129)	72 (60)	j331 (156)
Lewy bodies (any), n (%)	8 (17%)	9 (23%)	1 (25%)	3 (33%)
Amyloid angiopathy, n (%)	1 (2%)	h ^,^ j27 (68%)	0 (0%)	h ^,^ j7 (78%)
Gross infarcts, n (%)	11 (23%)	14 (35%)	1 (25%)	1 (11%)
Microscopic infarcts, n (%)	12 (26%)	13 (33%)	2 (50%)	1 (11%)

h Significantly different from Non-AD/Non-HS (*P*<0.05).

i Significantly different from AD (*P*<0.05).

j Significantly different from HS (*P*<0.05).

We also considered the possibility that the larger association of HS with hippocampal volume (compared to that of AD with hippocampal volume) could have been due to the cases of HS being pathologically more severe than the cases of AD encountered in this study (see cognitive scores of HS versus AD subjects, Table 6). We conducted further analyses to investigate this. In the first approach, we added to the ANCOVA model a cognition term (either MMSE score, global cognition score, or episodic memory score), intended to serve as a proxy for pathologic severity of either HS or AD. The ANCOVA results were similar regardless of which score was used as the cognition term. Specifically, the association of HS with volume remained significantly larger than the association of AD with volume. For the second approach, in order to simulate the groups of HS and AD subjects having the same mean cognitive scores (cognition was again used as a proxy for pathologic severity of HS and AD), the postmortem hippocampal volume measurements of AD subjects were extrapolated, based on the observed relation between hippocampal volume and measures of cognition in AD subjects. This correction factor accounted for a maximum of 0.18 cm^3^, or approximately 25%, of the difference in hippocampal volume between HS and AD subjects. Finally, in the third approach for equalizing the pathologic severity of the HS and AD groups, only the most severe cases of AD, meeting the NIA-Reagan criteria for high likelihood of AD, were included in the ANCOVA. In this analysis, the association of hippocampal volume with AD became stronger (−0.43 cm^3^), as expected, but was still substantially weaker than the association of hippocampal volume with HS (−0.75 cm^3^) (*P* = 1.6×10^−7^).

### Hippocampal shape analysis in the presence of different neuropathologies

**Figure 4:**
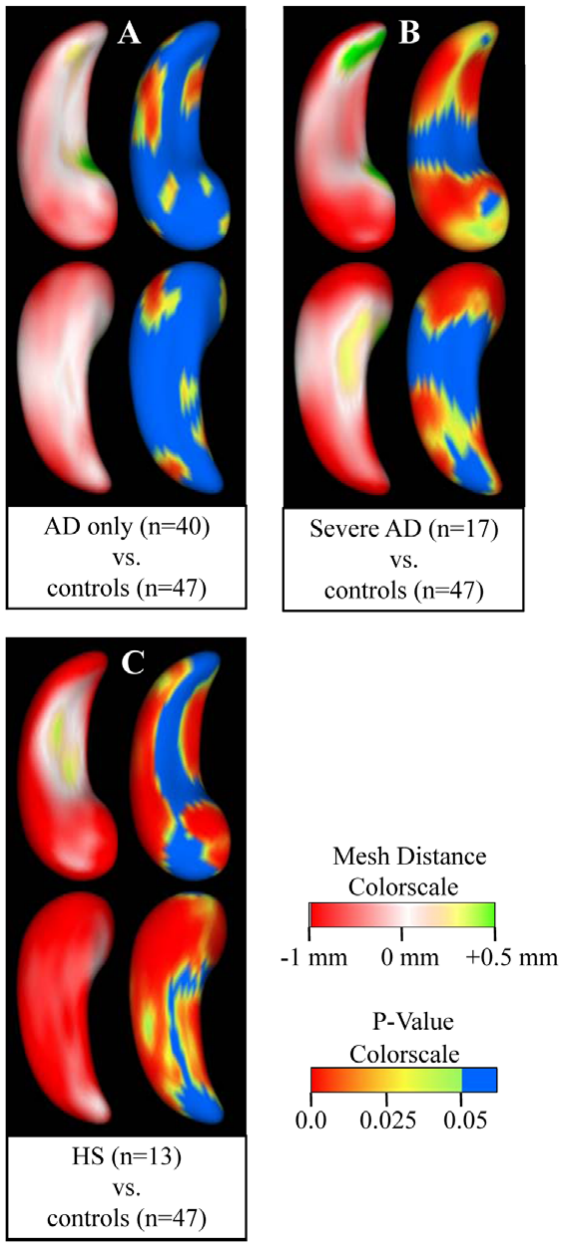
Shape analysis of (A) hippocampi affected by Alzheimer's disease (AD) and not hippocampal sclerosis (HS) compared to controls, (B) hippocampi most severely affected by AD compared to controls, and (C) hippocampi affected by HS with or without AD compared to controls. The top row of images contains the superior view of the hippocampus, while the bottom row contains the inferior view. Within each comparison, the left side shows a map of the difference in distance between points on the surface mesh of the first group versus the corresponding points on the surface mesh of the second group, projected along a unit vector that is normal to the surface. Thus, red shading shows areas of inward deformation of the first group relative to the second group, and green shading shows areas of outward deformation. The right side of each comparison shows significance maps with color indicating P-value. The significance maps show regions where points on the surface mesh of the first group are significantly displaced from the corresponding points on the surface mesh of the second group in any direction.

**Figure 5:**
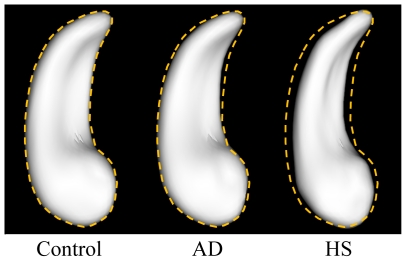
The average shapes of hippocampi affected by neither Alzheimer's disease (AD) or hippocampal sclerosis (HS) (N = 47), AD only (N = 40), and HS (N = 13, 9 with AD and 4 with HS only), viewed from the superior direction. The dashed line is the outline of the control hippocampal surface mesh.

For hippocampi affected by AD only (not HS), the difference in shape compared to hippocampi unaffected by either disease was greatest in the medial-lateral direction, primarily due to inward deformations of the surface near the head and tail of the hippocampus (Fig. 4A). When only the most pathologically severe cases of AD (meeting the NIA-Reagan criteria for high likelihood of AD) were included in the analysis, a similar pattern of shape difference was observed (Fig. 4B), but the inward deformations of the surface mesh were even larger in magnitude and significance. For hippocampi affected by HS (9 also with AD, 4 without AD), the surface mesh was deformed inward in excess of 1 mm at many locations throughout the structure, when compared to hippocampi unaffected by either condition (Fig. 4C). The superior surface of the hippocampal body was relatively preserved compared to other regions. The overall differences in shape between the control hippocampi, those affected by AD, and those affected by sclerosis are visible in Figure 5. Results were essentially the same when hemisphere volume was not accounted for in the shape analysis.

## Discussion

Postmortem MRI ensures that brain changes are minimized between imaging and histology, and therefore may be particularly useful in directly assessing the neuropathologic correlates of structural MRI changes in the hippocampus in the elderly. However, the value of the information collected with postmortem MRI has not been thoroughly assessed. Therefore, the current study first tested the hypothesis that hippocampal volume, when measured with MRI postmortem, is linked to clinical diagnosis of AD, as shown by studies that employed in vivo (antemortem) MRI. We then tested the hypothesis that hippocampal volume measured postmortem is positively related to antemortem cognitive performance, as shown by in vivo MRI studies. After these validation steps, we proceeded to use postmortem MRI and histology in an investigation of the neuropathologic correlates of structural MRI changes in the hippocampus in the elderly.

Results showed that hippocampal volumes measured postmortem were significantly lower in clinically diagnosed AD patients compared to MCI or NCI subjects, consistent with hippocampal atrophy in clinically diagnosed AD patients. Similar results were presented in most previously published reports that employed MRI volumetry of the human hippocampus in vivo [2], [6], [8], [9], [14], [43]. The current work also indicated that the hippocampal volume for MCI subjects was not significantly different than that of NCI subjects. However, other published research measured significant differences in hippocampal volumes between living subjects with normal cognition and those with MCI or questionable dementia [8], [22], [23], [44], [45]. This discrepancy may be due to one or more of the following factors: differences in the sample sizes; differences in ROI selection techniques; differences in spatial resolution of the MR images; the fact that the MCI group of the present study was not restricted to cases of amnestic MCI; or the fact that in the present study hippocampal volume was not normalized with intracranial volumes, but instead total hemisphere volume or height were separately included in the models as a covariate.

Significant positive relationships between hippocampal volume and the z-scores in five cognitive domains (episodic, semantic, and working memory, perceptual speed, and visuospatial ability, Table 3), as well as in the global cognition domain, were revealed when each domain was examined separately. Similar findings were obtained in previously published studies that demonstrated linkages between in vivo hippocampal volume and a measure of working memory [17], the Wechsler memory scale, and performance in the California verbal learning test and the Grober and Buschke test [11].

The scores for the five cognitive domains investigated here are known to have a high degree of correlation with each other. Therefore, the use of a combined regression model was necessary. When the z-scores from all five cognitive domains were included in a combined linear regression, hippocampal volume was shown to be significantly related only to episodic memory. Although in younger subjects the association of hippocampal volume with episodic memory is tenuous, many studies involving elderly subjects have reported a positive and significant relation. This is possibly because hippocampal volume and episodic memory both tend to erode in later life [24]. The positive relation of hippocampal volume to episodic memory found in the current work is consistent with previous findings since the current study involved very old subjects.

The finding that hippocampal volume is more strongly related to episodic memory than other cognitive domains is also consistent with existing knowledge of hippocampal function. Episodic memory is very closely linked to the hippocampus, even more so than semantic memory [46], [47]. Contributions to working memory come not only from the hippocampus, but also from other brain regions [48], [49]. Similarly, perceptual speed [50] and visuospatial ability [51] have not been wholly attributed to the hippocampus, so these cognitive domains may not be as strongly related to hippocampal volume as is episodic memory. As shown by the association of smaller hippocampal volume with clinical diagnosis of AD, and also by the relation of hippocampal volume to cognition (especially in the episodic memory domain), hippocampal volume measured via postmortem MRI captures important information related to the disease state of the brain.

It was demonstrated that AD pathology had a large and significant relation to hippocampal volume (Table 5). Other studies have employed postmortem MRI to examine the relation between AD pathology and structural changes in the MTL; however, these studies either had a relatively small number of subjects [13], [15], did not investigate other common pathologies that may affect hippocampal volume [14], [17], or lacked a quantitative measure of hippocampal volume [16]. The current study is unique in that it included a relatively large number of subjects (100) and showed that hippocampal volume measured using postmortem MRI was smaller in cases of pathologic AD, even after controlling for other common neuropathologies of aging.

The number of neurons in the hippocampus is correlated with its volume, both in control subjects and AD patients [13], [52]–[54]. Therefore, neuronal loss is a plausible etiology of the AD-related reduction in hippocampal volume observed in this study. Substantial evidence points to an apoptotic mechanism of cell death in AD [55], [56], possibly spurred on by amyloid beta peptide toxicity [57]. A necrotic pathway of cell death may also be involved [58]. Other plausible etiologies of hippocampal atrophy include loss of axons and dendrites, loss of non-neuronal elements such as glia and vessels, or other phenomena occurring in association with neurodegeneration and other age related pathologies.

HS was shown to have an even larger association with hippocampal volume than AD pathology. However, based on cognitive scores, the 13 HS subjects in the current study were more cognitively impaired than the AD subjects. It follows that the cases of HS may have been more severe pathologically than the cases of AD. Therefore, we separately employed three approaches to account for the potential difference in the pathologic severity of HS and AD cases in this study – first by including a cognitive score in the ANCOVA as a proxy for pathologic severity; second by extrapolating the hippocampal volumes of AD subjects based on their cognitive scores, again using cognition as a proxy for pathologic severity; and third by including only the most severe cases of AD in the analysis. All approaches yielded essentially the same finding, namely that HS has a substantially greater association with postmortem hippocampal volume than does AD, even when the pathologic severity of the HS and AD groups are equalized to the extent possible.

It has been reported that the effect of HS on hippocampal volume is larger in subjects with lower amounts of AD pathology compared to those with higher amounts of AD pathology [2], but results of the current study do not support this finding, since the interaction factor between AD and sclerosis was not found to be significantly different from zero when it was included in the ANCOVA model. Furthermore, hippocampal volume was nearly identical between groups of subjects with HS only and those with HS and concomitant AD pathology (1.87 cm^3^ and 1.85 cm^3^, respectively). However, the current study included only 4 HS subjects that did not have concomitant AD pathology, which limited the statistical power of this comparison.

When examined in separate models, Lewy bodies and multiple microscopic infarcts showed marginally significant associations with reduced hippocampal volume, while severe amyloid angiopathy showed a significant association. However, none of these associations were found to be statistically significant in a combined model that included multiple neuropathologies. A weak effect of amyloid angiopathy on hippocampal volume has been reported [2]. Another study reported an association of smaller hippocampal volume with Lewy bodies, but the association of volume with AD was greater [59]. There are several possible explanations for these minor discrepancies. First, there were differences in statistical power across studies (Lewy bodies were only found in 21 of the 100 hemispheres in the current study, for example). Second, there may have been differences in the methods used for quantification of neuropathology and ROI selection, as well as differences in spatial resolution in the MR images. Third, the use of antemortem MRI in the referenced studies [2], [59] may have allowed for brain changes to occur between imaging and histology. Finally, the use of hippocampal volume measurements that were normalized with intracranial volumes in the referenced studies [2], [59] is different from the normalization technique used in the current study.

The current study used shape analysis in order to visualize the pattern of hippocampal shape in elderly subjects that have been histopathologically evaluated. Several studies have used shape analysis in living subjects who were clinically diagnosed with AD or MCI. One such study found a link between inward deformation of the hippocampal surface, primarily in the CA1 subregion, and diminished delayed recall ability in MCI and AD subjects [60]. Other studies showed that subjects with AD-type dementia had greater atrophy primarily, but not exclusively, in the CA1 subregion compared to non-demented controls [61]–[63]. It has also been shown that this inward variation of the hippocampal surface in the CA1 subregion potentially occurs early enough to predict a non-demented individual's likelihood of converting to AD-type dementia [64]. In the current study, the observed hippocampal shape in histopathologically confirmed cases of AD was similar to the results of the aforementioned studies that relied only on clinical diagnostic information for classification of subjects. The statistically significant inward deformation that was detected on the lateral side of the hippocampus and in the hippocampal head and tail (Fig. 4A and 4B) probably corresponds to volume loss primarily in the CA1 subdivision, consistent with the high level of AD pathology that is frequently found in this particular region [52], [65], [66]. Although AD pathology can be found in all subdivisions of the hippocampus depending on the stage of the disease, the fact that CA1 is affected first and most predominantly [65], [66] is probably the reason why neuroimaging studies that employ hippocampal shape analysis most commonly detect volume loss in CA1 [60]–[63].

The observed pattern of inward deformation of the surface mesh associated with HS is also consistent with existing knowledge of that condition. We observed large inward deformations along the medial and inferior surfaces of the hippocampal body (Fig. 4C), approximately corresponding to the CA1 subregion and subiculum, frequently reported as exhibiting the highest degree of neuronal loss and gliosis in HS [18], [19]. Furthermore, the CA2 and CA3 subregions are typically spared of sclerotic pathology [19], and in the current study, the relatively preserved region on the superior surface of the hippocampus appears to be at the approximate location of CA2 and CA3 (Fig. 4C).

It was not the goal of this study to systematically investigate the effectiveness of hippocampal volume or shape as an early biomarker of AD. Nevertheless, results show that raw hippocampal volume cannot be used alone as a tool for early diagnosis of AD, given that HS is also associated with hippocampal volume reduction. Imaging of brain structure (e.g. hippocampal shape), function, and physiology may need to be combined to achieve accurate AD diagnosis during life.

The current study has certain limitations. First, intracranial volumes were not available for hippocampal volume normalization; instead, hemisphere volume was included as a covariate in the models. As a result of this practice, it is conceivable that reductions in hippocampal volume may have been masked by proportional decreases in total hemisphere volume. For this reason, we repeated the entire analysis without accounting for hemisphere volume and found that the results were essentially unchanged. We also repeated the analysis with subject height replacing hemisphere volume as a covariate in the models [24], and again found no substantial differences in the results. Notably, AD and HS were still the only two types of pathology that showed a significant association with hippocampal volume. A second limitation of the current study stems from the fact that subjects with histories of epilepsy or seizures were not specifically excluded from the group of 13 subjects with HS. The concern lies in the possibility that idiopathic HS observed in elderly subjects represents a different pathological phenomenon than HS accompanied by seizures. Four of the 13 subjects had taken anti-convulsive medication at some point in their lives, though we have no record of epilepsy diagnosis.

This study demonstrated that, despite the tissue changes that occur as a result of extraction of the brain from the cranium, tissue decomposition, and fixation, and also despite the lack of intracranial volume measurements for volume normalization purposes, postmortem MRI volumetry of the human hippocampus captures useful information about the antemortem cognitive, clinical, and neuropathological state of brain tissue. Therefore, postmortem MRI in combination with histology may allow direct assessment of the neuropathologic correlates of hippocampal volume in the elderly. Specific findings revealed a statistically significant link between reduced postmortem hippocampal volume and clinical diagnosis of AD, in agreement with similar studies that employed traditional antemortem MRI. Positive relationships were also detected between hippocampal volume measured postmortem and antemortem cognition, particularly in the episodic memory domain, which is known to be highly associated with hippocampal function. Of the neuropathologies that were investigated, AD and HS had the strongest association with hippocampal volume. The hippocampal shapes related to AD and HS were consistent with existing knowledge regarding the hippocampal regions susceptible to volume loss in those diseases. Future investigations will utilize different types of MR contrast to extract additional information from postmortem brain tissue collected in the MAP and ROS longitudinal studies of aging.
